# Gp-100 as a Novel Therapeutic Target in Uveal Melanoma

**DOI:** 10.3390/cancers13235968

**Published:** 2021-11-27

**Authors:** Daniel Martinez-Perez, David Viñal, Isabel Solares, Enrique Espinosa, Jaime Feliu

**Affiliations:** 1 Department of Medical Oncology, Hospital Universitario La Paz, Paseo de la Castellana 261, 28046 Madrid, Spain; dmartinezperez@salud.madrid.org; 2 Department of Internal Medicine, Hospital Universitario 12 de Octubre, 28041 Madrid, Spain; isabel.solares@salud.madrid.org; 3 Department of Medical Oncology, Hospital Universitario La Paz, IdiPAZ, Catedra UAM-AMGEN, Centro de Investigación Biomédica en Red Cáncer (CIBERONC), 28046 Madrid, Spain; enrique.espinosa@salud.madrid.org (E.E.); jaime.feliu@salud.madrid.org (J.F.)

**Keywords:** uveal melanoma, tebentafusp, glycoprotein 100

## Abstract

**Simple Summary:**

Glycoprotein 100 (Gp-100) is a protein highly expressed in melanoma tissue that has recently been effectively targeted by tebentafusp, a first-in-class bispecific protein of the immune-mobilizing monoclonal T cell receptors against cancer (ImmTACs) family. Recently, a randomized phase III trial reported an overall survival benefit for tebentafusp in patients with untreated metastatic uveal melanoma.

**Abstract:**

Uveal melanoma is a rare neoplasm with poor prognosis in the metastatic setting. Unlike cutaneous melanoma, treatment with kinase inhibitors or immune checkpoint inhibitors is not effective. Glycoprotein 100 (Gp-100) is a protein highly expressed in melanocytes and melanoma that has recently been effectively targeted by tebentafusp, a first-in-class bispecific protein of the immune-mobilizing monoclonal T cell receptors against cancer (ImmTACs) family. Tebentafusp targets tumor cells that express a peptide of Gp-100 presented by HLA*A0201, creating an immune synapse that kills targeted tumor cells. Recently, a randomized phase III trial reported an overall survival benefit for tebentafusp in patients with untreated metastatic uveal melanoma. The aim of this comprehensive review is to summarize evidence of Gp-100 as a therapeutic target in melanoma, and the preclinical and clinical development of tebentafusp as a novel therapeutic strategy for patients with uveal melanoma.

## 1. Introduction

Uveal melanoma (UM) is a rare neoplasm that arises from uveal melanocytes [1] and represents 3–5% of all melanomas. The incidence of UM varies across countries, from 1 to 9 per million populations per year, and increases in countries with higher latitudes in the northern hemisphere [2]. Unlike cutaneous melanoma (CM), nearly all UMs harbor a mutation in the Gαq pathway, mainly GNAQ and GNA11, both of which are α subunits of G proteins [1]. Another significant difference between UM and CM is the very low tumor mutational burden of the former, which may, to some extent, explain the lower efficacy of immunotherapy in UMs [3]. The prognosis of UM is dismal, as 50% of patients with a UM diagnosis eventually develop metastases [4]. Chemotherapy was considered the standard of care for patients with stage IV disease, but the response rates are generally under 10% [5]. Recently, the combination of immunotherapy with nivolumab and ipilimumab obtained positive results in a phase II trial, and replaced chemotherapy as the treatment of choice [6,7]. However, the median overall survival (OS) is still around 12 months [8].

Glycoprotein 100 (Gp-100) is a transmembrane glycoprotein, highly expressed in normal melanocytes and melanoma cells [9]. Several approaches have been developed to exploit its immunogenic potential, such as vaccines, adoptive cell therapy, and a new class of molecules, named ImmTACs.

Tebentafusp is a first-in-class anti-Gp-100 immune-mobilizing monoclonal T cell receptor against cancer (ImmTAC), a novel form of immunotherapy. In a recently published international phase III clinical trial, patients with UM, who received tebentafusp, had significantly prolonged median OS compared to patients in the control treatment group [10]. The aim of this comprehensive review is to summarize the evidence of Gp-100 as a therapeutic target in melanoma, and the preclinical and clinical development of tebentafusp as a novel therapeutic strategy for patients with UM. 

## 2. Materials and Methods

To identify the most relevant information about Gp-100, tebentafusp, and UM, we performed a systematic search in electronic databases, including PubMed, Embase, Cochrane, studies registered in clinicaltrials.gov, accessed on 29 August 2021, and publications from major international oncology meetings (American Society of Clinical Oncology Annual Meeting and the European Society for Medical Oncology (ESMO) Congress) until September 2021. Publications were searched using the following key terms: “Gp-100 and melanoma”, “Glycoprotein 100 and melanoma”, “tebentafusp and uveal melanoma”, and “ImmTACs and Gp-100”. We excluded papers that did not focus on the role of the Gp-100 protein in cancer, potential future therapies that have not yet been tested in humans, and titles without abstracts. Ninety-nine papers were initially identified, although, after applying the exclusion criteria, 37 were finally selected as relevant.

## 3. Discussion

### 3.1. Glycoprotein 100

Glycoprotein 100 (Gp-100) is a 100 kDa molecule specific to the melanocyte cells of the skin, mucosa, and retina. Gp-100 is a glycosylated transmembrane protein involved in melanosome maturation; which are the organelles that transport melanin. The RNA transcripts of Gp-100 are highly expressed in melanoma tissue at all stages, even in amelanotic lesions, whereas, in normal melanocytes, they are weakly detected [8]. Uveal and cutaneous melanoma differ in their Gp-100 tissue expression (80% of UM lesions express Gp-100 compared to 63% in CM) [11].

One of the first applications of Gp-100 was the development of antibodies, such as HMB-45, for the immunohistochemical diagnosis of melanoma. HMB-45 has good specificity for melanocytic lesions, although its sensitivity varies across different tumor stages (77–100% in primary melanoma cells and 69–93% in the metastatic setting) [12].

The first clinical evidence of Gp-100 as a potential therapeutic target for melanoma arose from the investigation of the immunological characteristics of cultured melanoma tumor-infiltrating lymphocytes (mTILs). Several studies established Gp-100 as a common immunogenic melanoma antigen; Kawakami et al. described that mTILs recognized short peptides presented by HLA-A2 restricted class I MHC molecules, such as YLEPGPVTA, an epitope of Gp-100 [13]. Moreover, a clinical trial exposed 20 melanoma patients to cultured autologous mTILs with interleucin-2, resulting in 30–40% objective clinical responses [14]. Eight out of these 20 mTILs recognized Gp-100 [15,16].

Different approaches targeting Gp-100 as an antigen for the treatment of melanoma have been developed, including Gp-100-based vaccines, mRNA electroporated dendritic cells, and a fusion protein denominated IMCGp-100, or tebentafusp.

### 3.2. Vaccines Targeting Gp-100 Peptides

Multiple Gp-100-related vaccines have been tested in clinical trials (NCT00094653, NCT00036816, NCT01989572). Gp-100:209-217 is the only peptide vaccine, evaluated in a phase III clinical trial, that has a statistically significant clinical benefit in patients with irresectable melanoma [17]. Patients were randomized in a 1:1 ratio to receiving interleukin-2 alone or with the vaccine plus incomplete Freund’s adjuvant (Montanide ISA-51). The median progression-free survival (PFS) was significantly longer in the vaccine–interleukin-2 group than in the interleukin-2 alone group (2.2 months vs. 1.6 months; *p* = 0.008), and there was a trend towards a longer median OS (17.8 months vs. 11.1 months, *p* = 0.06). The absence of a survival benefit, the approval of immune checkpoint inhibitors (ICIs) in the same scenario shortly after, and the toxicity of interleukin-2 has probably precluded the approval of this vaccine by regulators. 

### 3.3. Adoptive Cell Therapy Targeting Gp-100

The potential of Gp-100 as a tumor antigen is also being considered in the field of adoptive cell therapy. TriMixDC-MEL is a therapy based on mRNA electroporated autologous dendritic cells. These cells are created in a two-step process. First, mRNA is delivered into the DCs encoding three proteins (CD40L, CD70, and caTLR4). This results in the generation of functionally mature DCs that activate T cells, support their proliferation, and inhibit their apoptosis. Next, mRNA encoding the fusion of an MHC class II protein (DC-LAMP) with four melanoma antigens (Gp-100, MAGE-A3, tyrosinase, and MAGE-C2) is delivered [18]. Two phase II clinical trials have been published regarding the use of TriMixDC-MEL as an adjuvant therapy for patients with stage III/IV resected melanoma (showing a significant benefit 1-year disease-free survival of 71% in 21 patients receiving the experimental treatment vs. 35% in the control arm) [19], and in the palliative setting, combined with ipilimumab, for patients with metastatic melanoma (with an overall response rate of 38% and a 6-month disease control rate of 51%) [20]. No randomized clinical trial has demonstrated a significant clinical benefit.

### 3.4. Tebentafusp: Gp-100 Directed ImmTAC

Tebentafusp is currently the first and only drug of a new class of molecules called ImmTAC (immune-mobilizing monoclonal T cell receptors against cancer) that has demonstrated a clinical benefit in a phase III clinical trial [9]. Furthermore, tebentafusp is the only therapy targeting Gp-100 that has been granted the Food and Drug Administration (FDA) Breakthrough Therapy Designation [21] and has been admitted to an accelerated assessment by the European Medicines Agency (EMA).

#### 3.4.1. Preclinical Development of ImmTACs

ImmTACs consist of a modified T cell receptor (TCR) that detects a particular peptide bound to a specific human leukocyte antigen (denominated p-HLA complex) on the surface of cells. The TCR is combined with a single-chain antibody against CD3, which is a known co-receptor involved in activating T cells. Therefore, ImmTACs detect tumor cells that present a specific p-HLA, recruiting T cells to lyse the targeted cells [22].

**Figure 1:**
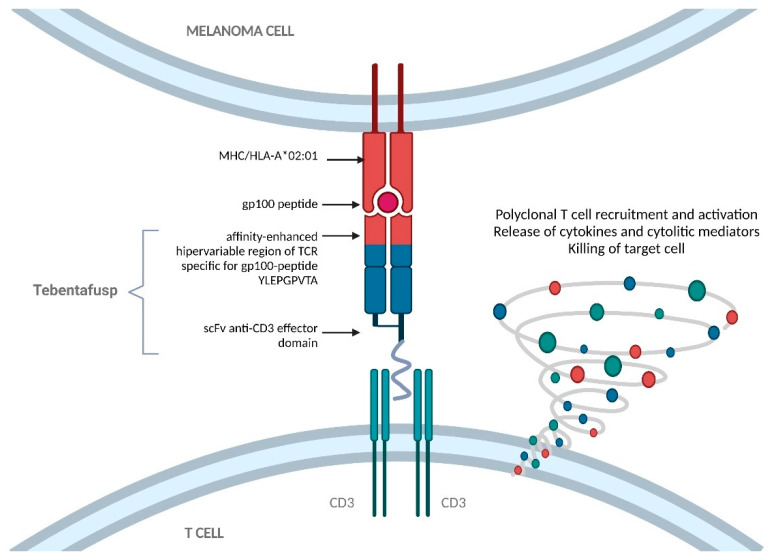
Structure and mechanism of action of tebentafusp. Tebentafusp comprises an engineered T cell receptor targeting an epitope of Gp-100 presented by HLA-A*02:01 cells, linked to an anti-CD3 single-chain variable fragment that can engage T cells. The interaction induces polyclonal T cell recruitment and activation, the release of cytokines and cytolytic mediators, and, ultimately, the killing of target cells. Gp-100, glycoprotein 100; HLA, human leukocyte antigen; MHC, major histocompatibility complex; TCR, T cell receptor.

Natural TCRs are transmembrane heterodimers that comprise two chains (alfa and beta) held together by a disulfide bond. The production of ImmTACs firstly required the development of stable and soluble TCRs by removing the transmembrane elements and adding an artificial disulfide bond [22]. The hypervariable regions of the TCR are mutated by directed molecular evolution and phase display selection to obtain the molecule with the highest affinity for the desired p-HLA, in the order of the picomolar range [23]. Finally, an anti-CD3 domain is added to the modified TCR, to simulate natural immune activation. This anti-CD3 fragment has a lower affinity (in the nanomolar range) to ensure that the T cells are not stimulated unless the ImmTAC has detected the desired target. ImmTACs ultimately result in the formation of a lytic immune synapse between T cells and the malignant cells [24] (Figure 1).

One of the main drawbacks of ImmTACs is that they must be developed by targeting a specific p-HLA complex. HLAs are part of a protein system that has an important role in the regulation of the immune system. Tebentafusp has been developed by targeting the most common HLA complex in humans, which is HLA-A*0201, although its prevalence is different across different ethnic groups. Fifty percent of Caucasians, 47% of Hispanics, and 35% of African Americans are HLA-A2 positive. HLA-A has several haplotypes (31 alleles have been described). HLA-A*0201 is the most prevalent. Ninety-six percent of HLA-A2 Caucasians present with HLA-A*0201, compared to 59% HLA-A2+ of African Americans, and 73% HLA-A2+ of Hispanics [25].

The following two ImmTACs, besides tebentafusp, are currently being tested in clinical trials: IMC-C103C and IMC-F106C [26]. IMC-C103C targets MAGE-A4 and is being tested in a phase I clinical trial (NCT03973333) against the following several solid tumors: synovial sarcoma, non–small-cell lung, gastric, head and neck, and ovarian cancer. IMC-F106C targets PRAME and is being tested in a phase I clinical trial (NCT04262466) in the following different tumors: breast, endometrial, ovarian, and small-cell lung cancer.

#### 3.4.2. Preclinical Development of Tebentafusp

The specific p-HLA complex detected by tebentafusp is YLEPGPVTA-HLA-A*0201, which is an immunogenic peptide derived from Gp-100. Cell lines with as few as 70 copies of the p-HLA complex have been detected by tebentafusp, manifesting its high sensitivity [23].

In vitro studies have shown that tebentafusp activates both CD8+ and CD4+ T cells by exhibiting a polyfunctional phenotype, which consists of the T cells expressing key molecules, such as CD-40, interferon-gamma, tumor necrosis alpha, and interleukin-2. This state is considered critical for anti-tumor responses in adoptive cell therapy [27]. There is evidence showing that tebentafusp not only activates T cells directly, but it also potentiates antigen cross-presentation by dendritic cells, which might have a role in a durable immune response [28]. 

The in vivo activity of tebentafusp has been reported in xenograft immunodeficient mice models transplanted with melanoma tumor cells expressing Gp-100. The mice exposed to tebentafusp concomitantly with human, non-stimulated peripheral blood mononuclear cells (PBMCs) showed significant tumor size reductions [23]. 

Activation of the immune system has also been demonstrated in humans. Patients who have been administered tebentafusp presented a significant elevation in chemokines, mainly CXCL10, with a median of 66-fold, compared to before administration [29]. CXCL10 is associated with a shift in Th1 and CD8 T cells from the blood to peripheral sites [30]. Biopsies comparing the tumor microenvironment pre and post tebentafusp administration have shown an increase in intratumoral CD8+ cells, even in patients with few intratumoral T cells prior to treatment [29].

#### 3.4.3. Clinical Development of Tebentafusp

The following three clinical trials have published results regarding the administration of tebentafusp in humans: IMCGp-100-01 [29], IMCGp-100-102 [31], and IMCGp-100-202 [9]. Except for IMCGp-100-01, which tested for both metastatic UM (mUM) and metastatic CM, phase II and III trials have only been performed in mUM. The design and outcomes of these trials are summarized in Table 1. A phase I/II clinical trial combining tebentafusp and ICIs is ongoing for patients with mCM (NCT02535078). 

The first-in-human study of tebentafusp [29] included 84 heavily pretreated mCM (*n* = 61), mUM (*n* = 19), and other origin (vulval, mucosal, and unknown) melanoma (*n* = 4) patients, who were administered weekly (arm 1) and daily (arm 2) doses of tebentafusp, with the primary objective of establishing its safety and tolerability. At the 900 ng/kg dose, two out of four patients experienced dose-limiting toxicity (DLT) of hypotension, establishing 600 ng/kg, or 50 µg as a flat dose, as the maximum tolerated dose (MTD). Efficacy was assessed in patients with RECIST evaluable lesions and who were treated with a dose of ≥270 ng/kg or 50 µg. A one-year OS rate of 65% and an overall response rate (ORR) of 8.7% were achieved, with a further 55% of the patients experiencing stable disease.

A phase II clinical trial [31], performed on 127 patients, included exclusively pretreated mUM patients. Tebentafusp was administered intravenously at a dose of 20 μg on day one, 30 μg on day eight, and 68 μg weekly thereafter. The ORR was 5%; the disease control rate (DCR), which included partial and stable responses, was 50%; the median duration of response was 8.7 months; the mOS was 16.7 months, with a one-year OS of 62%. These results were better than expected, according to previous trials on mUM patients [5,8]. 

IMCGp-100-202 [9] is an open-label, phase III trial, in which 378 HLA-A*0201 patients with untreated metastatic uveal melanoma were randomly assigned, in a 2:1 ratio, to tebentafusp or the investigator’s choice of therapy, including ICIs (94%) or dacarbazine (6%). Patients were excluded if they had symptomatic brain metastases or an autoimmune disease requiring immunosuppressive treatment. Patients were administered tebentafusp at the same dose as in the phase II trial, and were monitored overnight for the first 3 weeks. The primary endpoint was the OS. The secondary endpoints include disease control, response rate, and PFS. Preplanned subgroup analysis for the OS of patients with or without a rash was prespecified. 

This trial met its primary endpoint, achieving a 12-month OS of 73% in the intention-to-treat population in the experimental arm, and 59% in the control group. The median OS was 21.7 months (95% confidence interval (CI): 18.6 to 28.6) and 16.0 months (95% CI: 9.7 to 18.4), respectively, with a hazard ratio (HR) for death of 0.51 95% CI: 0.37 to 0.71; *p* < 0.001. The progression-free survival (PFS) at 6 months was higher among patients treated with tebentafusp, compared with the control group (31% vs. 19%, respectively). The median PFS was 3.3 (3.0 to 5.0) and 2.9 (2.8–3.0), respectively, with an HR of 0.73 (95% CI: 0.58 to 0.94; *p* = 0.01). The response rate, as per RECIST1.1, was low in both groups (9% and 5%, respectively); however, the disease control was higher in the experimental arm (46% vs. 27%, respectively). 

Survival benefit was observed across several prespecified subgroups, including patients with lactate dehydrogenase (LDH) above the upper limit of normal. Interestingly, patients with disease progression as the best response also had a significant benefit in OS (15.3 months in the experimental group vs. 6.5 months in the control group; HR for death 0.43, 95% CI 0.27–0.68), which raises the question of whether using classical radiological criteria for tumor response assessment is the most appropriate method for ImmTAC response evaluation. 

To address this issue, a sub-study of the IMCGp-100-102 trial of circulating tumor DNA (ctDNA) in patients with mUM treated with tebentafusp in the second-line setting was presented at the ESMO Congress 2021 [32]. Of the 127 patients included in the study, 116 (93%) had detectable ctDNA at the baseline, and 99 patients at baseline and by week 9. Seventy percent of the evaluable patients had a ctDNA reduction, despite a RECIST1.1 response rate of < 10%. Fourteen percent of patients had complete ctDNA clearance. Patients with a reduction in ctDNA ≥ 0.5 log had a significant OS benefit compared with those with a <0.5 log ctDNA reduction (HR, 0.56 (95% CI, 0.32–0.95); *p* = 0.03), irrespective of the RECIST response. Among the patients with progressive disease (PD) by RECIST1.1, ctDNA reduction was observed in 64%. Patients with PD and a reduction in ctDNA ≥ 0.5 log had a significant OS benefit compared with those with a <0.5 log ctDNA reduction (HR 0.44 (95% CI 0.2–0.94) *p* = 0.027).

Other associated survival prognostic factors described in early clinical trials, but not proven in the pivotal trial, are rash appearance, CXCL10 levels, and a decrease in circulating CXCR3+ CD8+ T cells [29,31]. Non-prespecified genomic analysis has revealed interesting associations with survival in the IMCgp100-102 clinical trial. Although GNAQ, GNA11, SF3B1, and tumor mutational burden (TMB) were not associated with survival, high baseline mRNA levels of the type 1 IFN inducible and ISGylation gene UBA7, high expression of the type 2 IFN inducible GTPase binding gene GBP1, and high baseline levels of JAK2/STAT4 were associated with tumor shrinkage and overall survival [33]. In contrast, previous treatment with ICIs does not modify the clinical benefit or on-target adverse events in patients with mUM treated with tebentafusp in the second-line setting [34].

#### 3.4.4. Toxicity Profile of Tebentafusp

Across several clinical trials, tebentafusp has proved to be a well-tolerated drug, with manageable side effects, and a low discontinuation rate (2–2.4%), due to drug-related toxicity across all the trials [9]. ImmTACs target a p-HLA complex, which might entail significant toxicities across non-tumoral cells expressing the same peptide in their MHC complexes. Gp-100 has the advantage of having differential expression between normal and tumoral melanocytes [11], which limits on-target off-tumor side effects.

Intrapatient dose escalation of tebentafusp decreased the rate of adverse events (AEs) in early phase trials. In the pivotal trial, patients received intravenous doses of 20 µg on day one, 30 µg on day eight, and 68 µg weekly thereafter. The incidence of AEs significantly decreased over time. At week one, 20% of the AEs were grade 3 or more, whereas, after week eight, the percentage of grade ≥ 3 AEs was less than 5%. Patients were closely monitored overnight for the first three weeks during dose escalation, to detect serious AEs, such as cytokine release syndrome (CRS). CRS is a potentially serious concern associated with T-cell engaging therapies, and has also been reported with tebentafusp. CRS with tebentafusp usually occurs within 24 h of administration, and is usually mild to moderate. Pyrexia (76%), chills (47%), and hypotension (38%) are the most common any-grade cytokine-related adverse events reported in the IMCGp-100-202 trial [9]. Grade ≥ 3 CRS was reported in < 5% of the patients, and could be managed with steroids and/or tocilizumab. No toxic deaths were recorded.

The most prevalent AE was rash (83% all grades, and 18% G3–4). Skin rash is an on-target, off-tumor event, as tebentafusp has been found to induce rapid recruitment of T cells in the proximity of intraepidermal melanocytes, and macrophage activation [35]. Grade ≥ 2 rash appearance has been linked to a significantly higher OS in early clinical trials, compared to no rash or grade 1 (HR 0.122, 95% CI (0.03, 0.45), *p* = 0.0015) [31]; however, in a prespecified analysis in the phase III clinical trial, no significant OS difference was found [7]. Other common side effects, such as pyrexia, pruritus, nausea, hypotension, and elevations of aminotransferases, were mild.

#### 3.4.5. Possible Mechanisms of Resistance to ImmTACs

ImmTACs are a novel therapeutic class of antitumoral drugs, and limited amount of data has been published in relation to the possible mechanisms of resistance, but several have been described in other T cell stimulation therapies, which might apply to this novel drug family [36]. The mechanism of resistance can be broadly divided into primary and acquired resistance.

Primary resistance is associated with early disease progression. It occurs when ImmTACs are unable to detect the specific p-HLA complex in vivo. As has been previously described [11,12], not all melanoma lesions express Gp-100 intracellularly, which might explain why a significant number of patients do not respond to this therapy. Another pattern of intrinsic resistance might be associated with an intense immunosuppressive tumor microenvironment, which might preclude the ImmTAC from activating T cells, even if they detect the desired p-HLA complex [36].

Acquired resistance is related to the loss of function of T cells by weakening their antitumor response [36]. A possible mechanism might be related to the upregulation of known inhibitory immune regulators, such as PD-1. The duplication of PD-L1 expressions in human melanoma tumor biopsies after administration of tebentafusp has already been described [31]. Preclinical studies have found that exhausted PD1+ TILs exposed to ImmTAC effectively kill PD-L1- tumor cells, but have reduced killing activity against PD-L1+ tumor cells. When pembrolizumab was administered, the killing activity was improved against these PD-L1+ cells [37]. An ongoing clinical trial (NCT02535078) combining ICIs and ImmTACs might shed some light on this issue [37]. Another possible mechanism of resistance might be related to the loss of the p-HLA complex in certain tumoral subclones. Gp-100 expression is not diminished after tebentafusp administration, at least in the first two weeks after treatment [31], but no data in relation to the loss of expression in later moments of the evolution of the disease have been published. 

## 4. Conclusions

Patients with uveal melanoma have a dismal prognosis in the metastatic setting, where no effective systemic treatments have previously been available. Tebentafusp is an ImmTAC directed to a Gp-100 peptide united to HLA-A*0201 that prolongs overall survival in patients with mUM. However, several challenges need to be addressed, such as the HLA-restricted eligibility of the patients, and the best tool to assess the tumor response and efficacy, to better understand the mechanism of resistance, and to test ImmTACs in combination with other therapies to enhance their potential benefit. Further investigation is warranted for this novel and promising treatment approach.

## Figures and Tables

**Table 1:** Design and outcomes of clinical trials with tebentafusp for uveal melanoma.

Clinical Trial	Design	Disease and N	DCR and ORR (%)	PFS	OS
IMCGp-100-01 [27]	Phase I	mUM (*n* = 19) mCM (*n* = 61)	57% and 14% 18% and 6%	-	mOS: 33.4 months 1Y-OS: 65%
IMCGp-100-102 [29]	Phase I cohort	heavily pretreated mUM *n* = 19	31% and 10%	1Y-PFS: 66%	mOS: NR 1Y-OS: 74%
	Phase II cohort	pretreated (1 line) mUM *n* = 130	50% and 5%	2.8 months	mOS: 16.8 months 1Y-OS: 62%
IMCGp-100-202 [7]	Controlled phase III	untreated mUM (*n* = 378) T (*n* = 252) vs. ICOT (*n* = 126)	T: 46% and 9% ICOT: 27% and 5%	3.3 vs. 2.9 months (HR, 0.73; 95% CI, 0.58–0.94)	21.7 vs. 16.0 months (HR, 0.51; 95% CI, 0.37–0.71)

DCR, disease control rate; HR, hazard ratio; ICOT, investigator’s choice of therapy mCM, metastatic cutaneous melanoma; mUM, metastatic uveal melanoma; ORR, overall response rate; OS, overall survival; PFS, progression-free survival; T, tebentafusp; Y, year.
